# Supplementary material for: Anion effects govern efficiency of electrochemical amine-mediated CO2 capture/release
Source: Nat Commun. 2025 Dec 11;17:489. doi: 10.1038/s41467-025-67177-7 (PMC12804695; doi:10.1038/s41467-025-67177-7)
Supplement: Supplementary file 1 — Supplementary Information [file 41467_2025_67177_MOESM1_ESM.pdf]

Supplementary Information

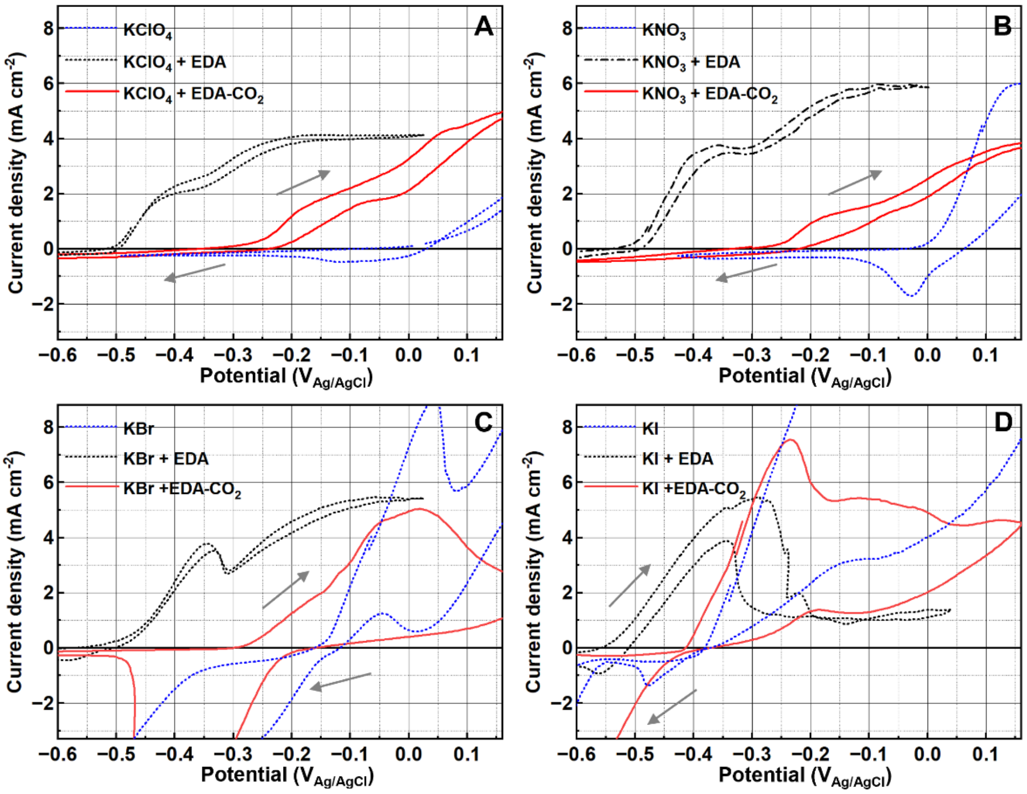

Fig. S1 CO_2 release-Cyclic Voltammetry for Cu foil in supporting electrolyte of 0.1 M (A) $KClO_4$, (B) KNO_3, (C) KBr and (D) KI in absence and presence of pure EDA and EDA-CO_2 carbamates in H cell.

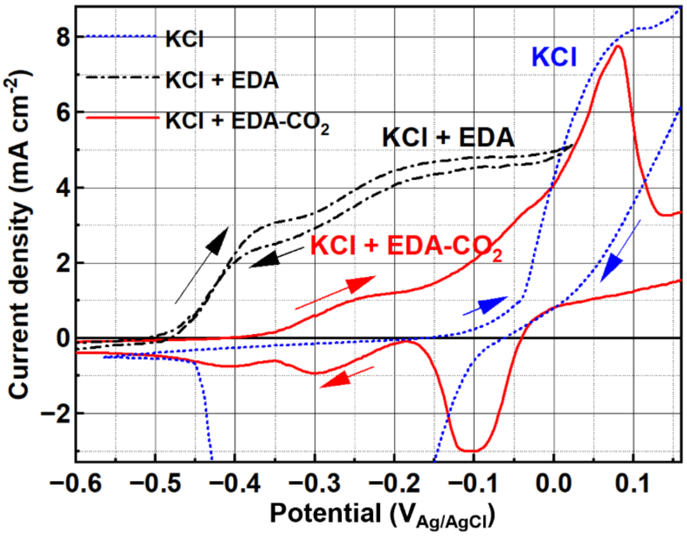

Fig. S2 CO_2 release-Cyclic Voltammetry for Cu foil in 0.1 M KCl, KCl with EDA, and KCl with EDA-CO_2, respectively in H cell.

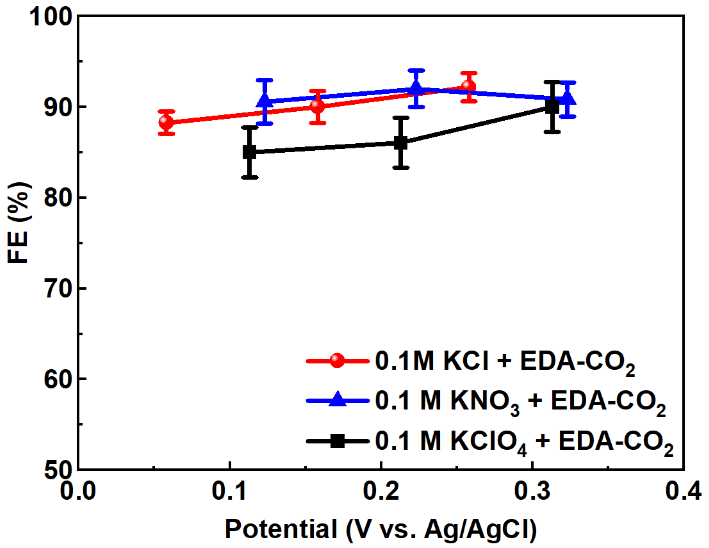

Fig. S3 Faradaic efficiency for Cu foil in different supporting electrolytes. Error bars represent the ± s. d. between triplicate measurements.

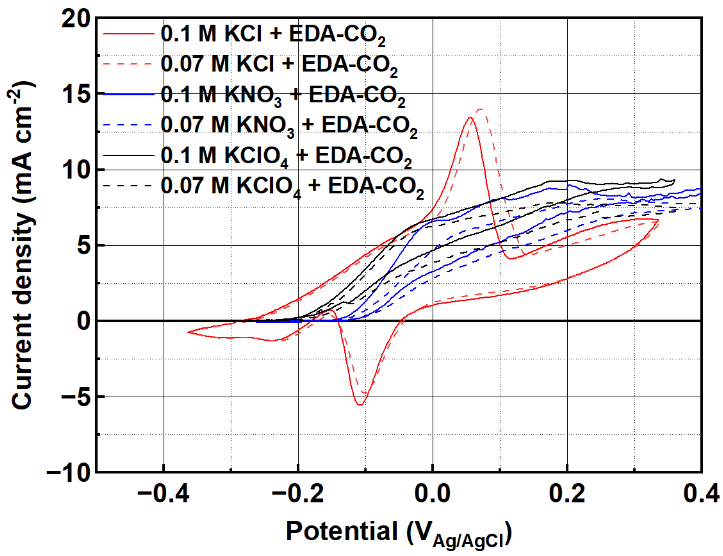

Fig. S4 Cyclic voltammetry of Cu foil in 0.1 M and 0.07 M KA (A = Cl^-, NO_3^-, and ClO_4^-) supporting electrolytes containing 0.1 M EDA–CO_2 carbamates. All the CVs were recorded at 5 mV s^{-1}.

0.1 M and 0.07 M supporting electrolytes were used for the measurements, and the anion concentration showed only a minimal impact on the CO_2 release behavior.

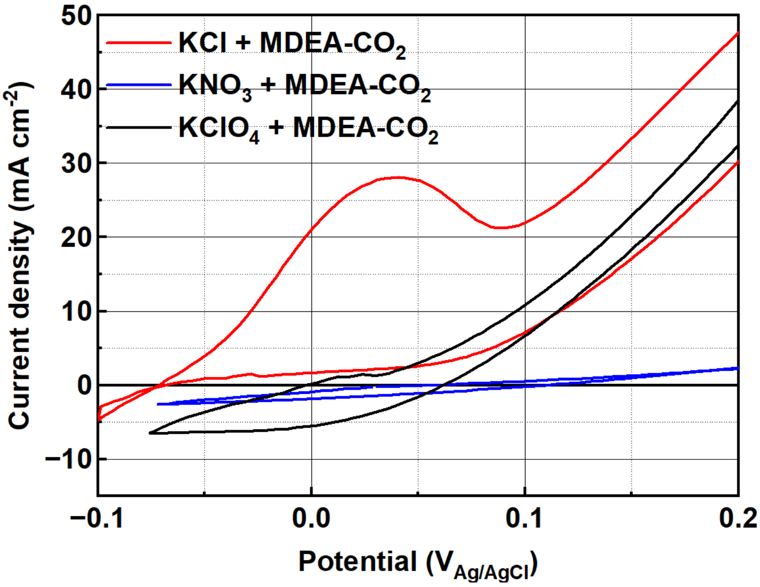

Fig. S5 CO_2 release-cyclic voltammetry for Cu foil in 0.1 M KA (A = Cl^-, NO_3^- and ClO_4^-) supporting electrolyte with 0.1 M N-methyldiethanolamine (MDEA)-CO_2 carbamates in H cell. All the CVs were recorded at 5 mV s^{-1}.

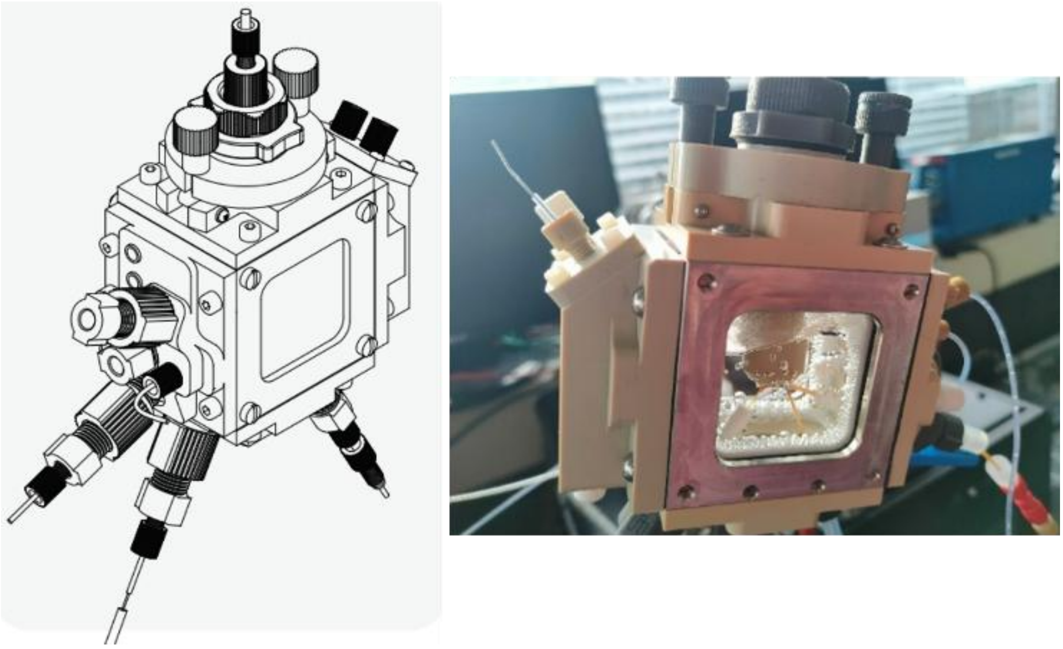

Fig. S6 Schematic illustration of custom-made capillary flow cell.

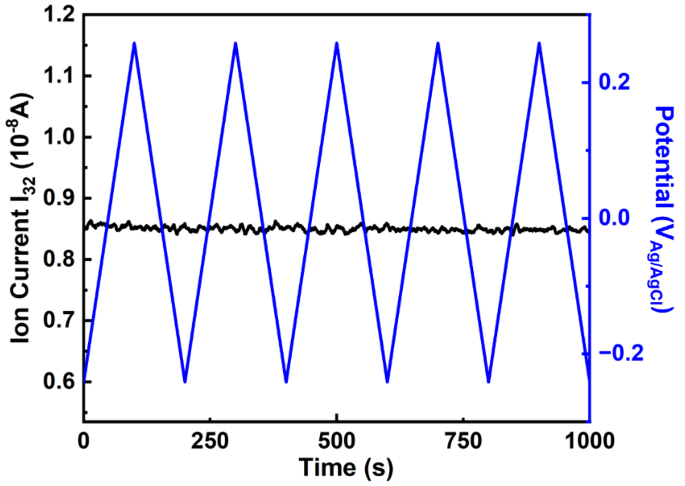

Fig. S7 DEMS signal of O_2 revealing CO_2 release on Cu electrodes as a function of applied potential.

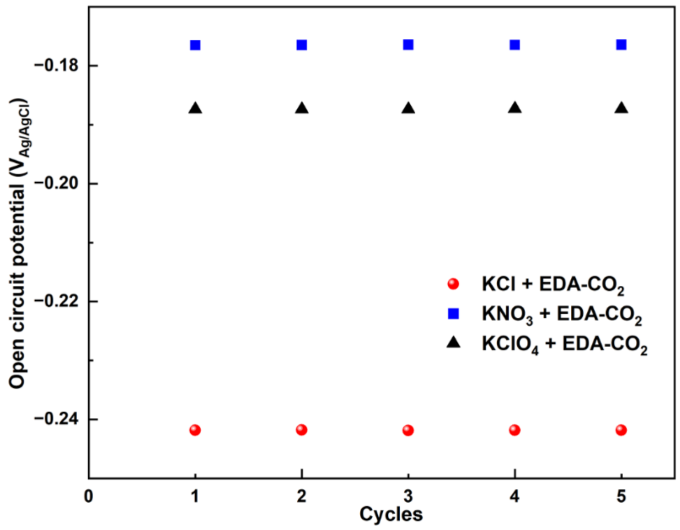

Fig. S8 The open circuit potential in various supporting electrolytes was measured using a Potentiostat during the operation of the DEMS capillary flow cell system.

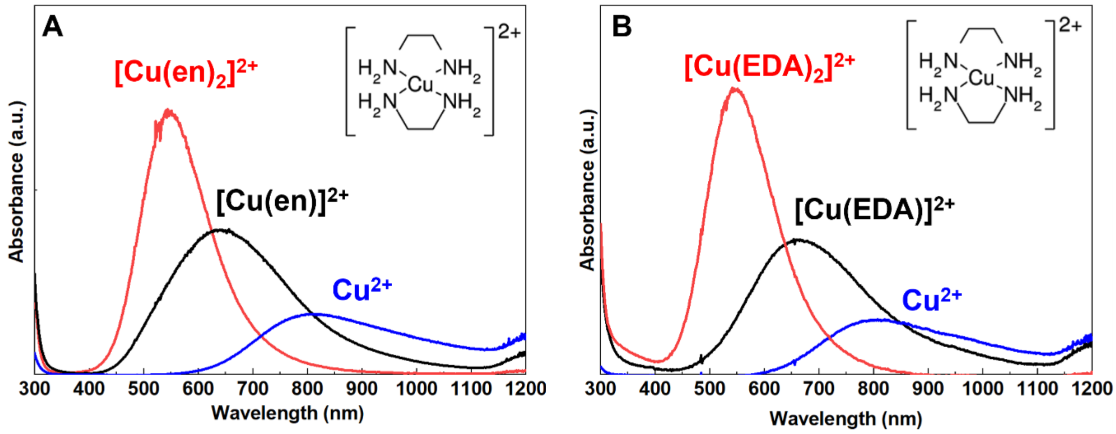

Fig. S9 UV-Vis spectra for Cu^{2+}, $[Cu(EDA)]^{2+}$, and $[Cu(EDA)_2]^{2+}$, respectively in (A) Cu-EDA and (B)Cu-EDA-CO_2 system.

UV-vis spectra showed absorption bands at 820, 650, and 550 nm in Cu-EDA and Cu-EDA-CO_2 system, respectively, which could be attributed to Cu^{2+}, $[Cu(EDA)]^{2+}$, and $[Cu(EDA)_2]^{2+}$, respectively.

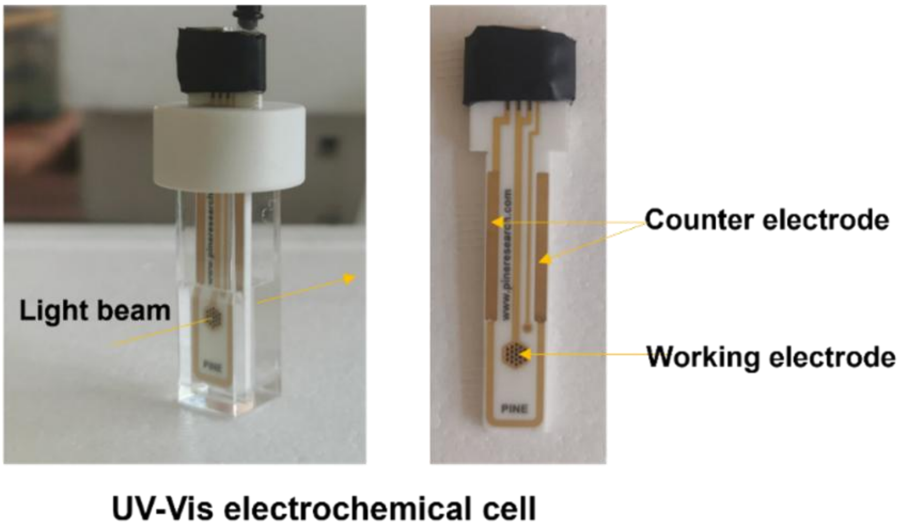

Fig. S10 Schematic illustration of spectro-electrochemical configuration. A patterned "honeycomb" electrode that mounts in a thin-layer quartz cuvette, with a secure cap ensuring precise alignment of the electrode and Ag/AgCl reference. The honeycomb electrode chip contains an onboard working and counter electrode.

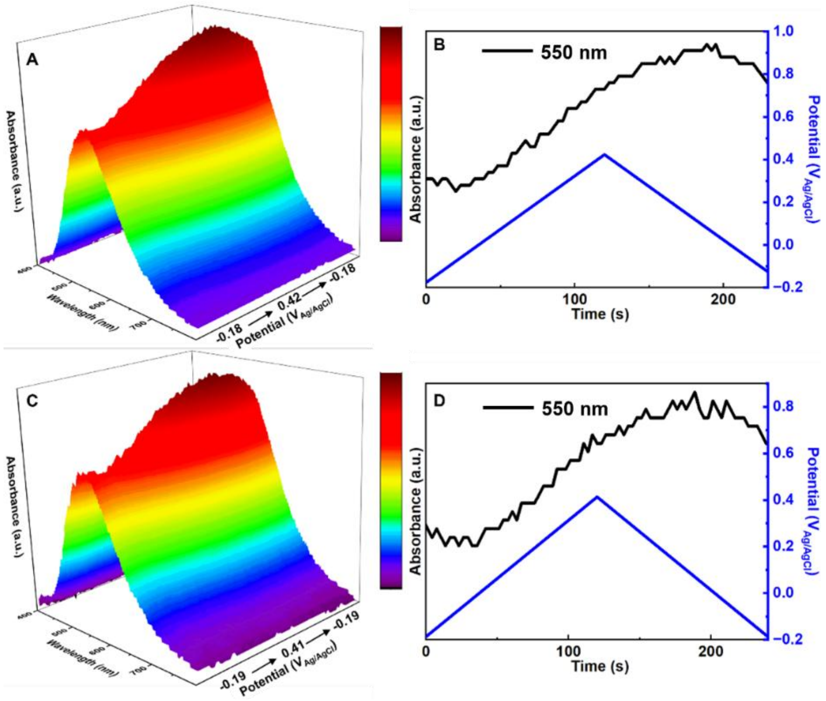

Fig. S11 (A) in situ UV-Vis revealing CO_2 release on Cu NPs as a function of applied potential in 0.1 M KNO_3. (B) Normalized peak area of CO_2 release signals on Cu NPs obtained from in situ UV-Vis in (A). (C) in situ UV-Vis revealing CO_2 release on Cu NPs as a function of applied potential in 0.1 M $KClO_4$. (D) Normalized peak area of CO_2 release signals on Cu n NPs obtained from in situ UV-Vis in (C).

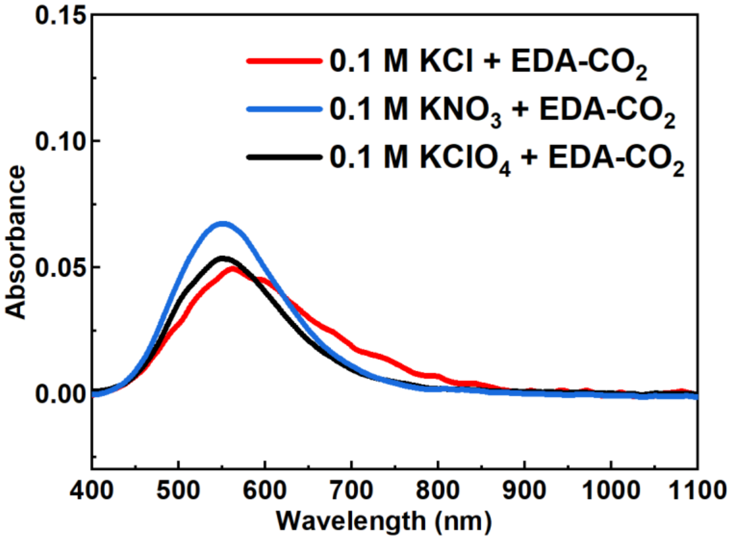

Fig. S12 In situ UV-Vis on Cu NPs in different electrolyte after CO_2 release.

In KCl + EDA-CO_2 system, $[Cu(EDA)_2]^{2+}$ saturates faster compared to the KNO_3 + EDA-CO_2 and $KClO_4$ + EDA-CO_2 systems, as evidenced by the higher absorbance intensity and the formation of $[Cu(EDA)]^{2+}$ near the surface of Cu NPs, given the same amount of Cu NPs on working electrode in all three systems.

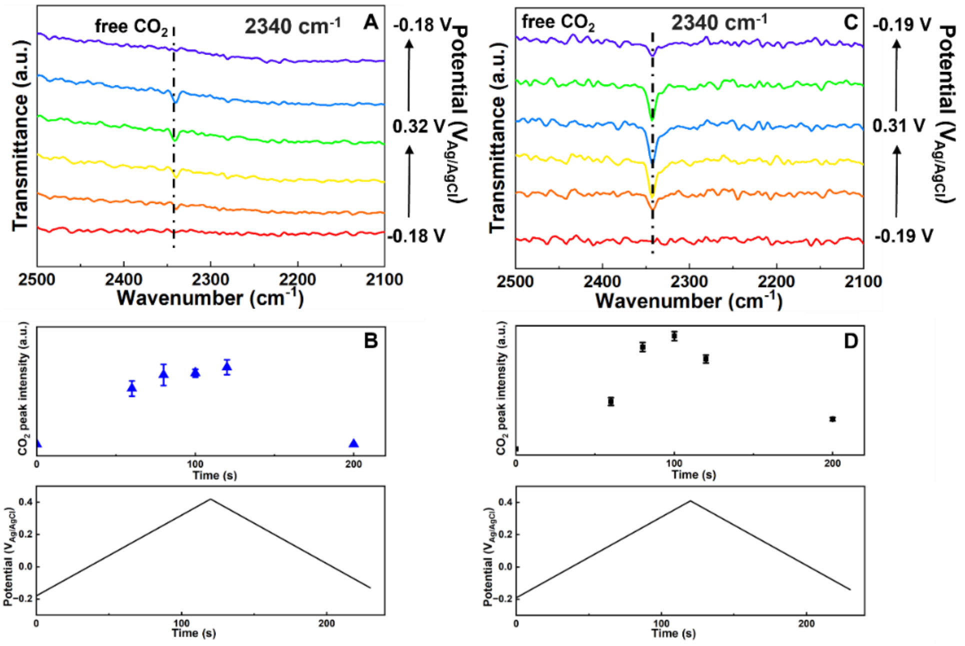

Fig. S13 (A) in situ FTIR revealing CO_2 release on Cu NPs as a function of applied potential in 0.1 M KNO_3. (B) Normalized peak area of CO_2 release signals on Cu n NPs obtained from in situ FTIR in (A). (C) in situ FTIR revealing CO_2 release on Cu NPs as a function of applied potential in 0.1 M $KClO_4$. (D) Normalized peak area of CO_2 release signals on Cu NPs obtained from in situ FTIR in (C). Error bars represent the ± s. d. between triplicate measurements.

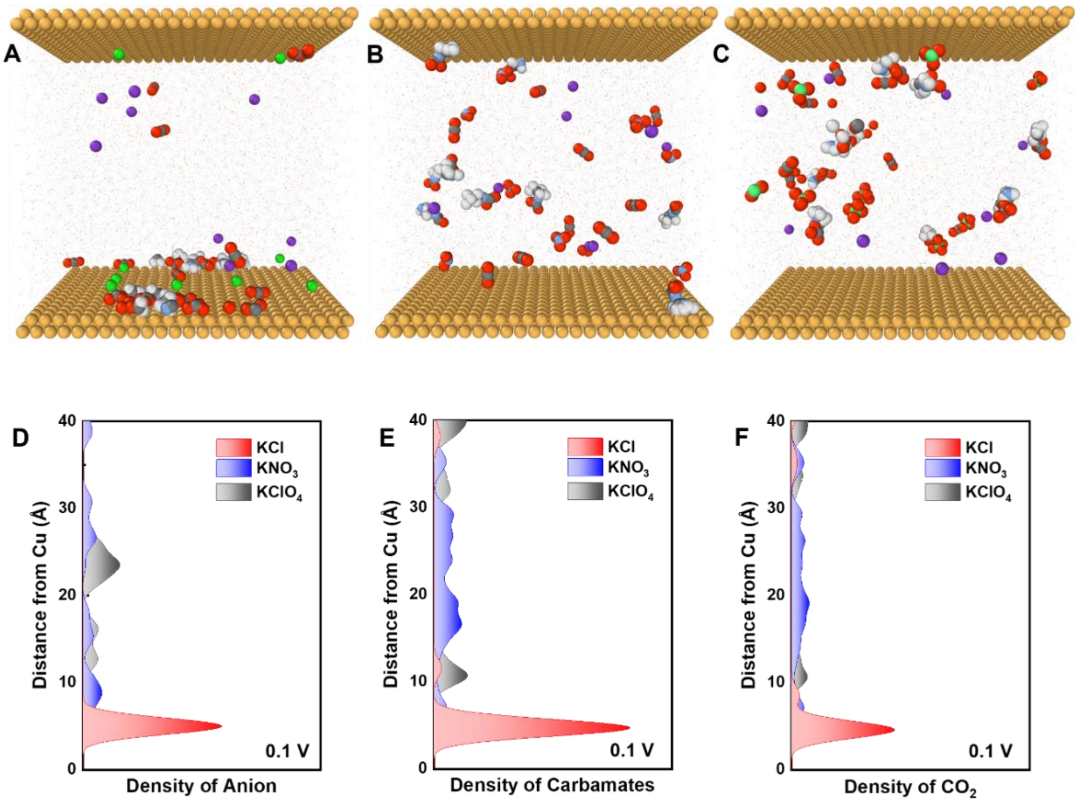

Fig. S14 Computational studies of the anion impact on EMAR interfacial CO_2 release dynamics. (A) Molecular dynamics predictions of atomic configurations for the (A) Cu-carbamates-Cl interface, (B) Cu-carbamates-nitrate interface, and (C) Cu-carbamates-perchlorate interface at the fixed potential (ΔU) of 0.1 V. Comparison of density distribution of (D) anions, (E) carbamates and (F) CO_2 molecules versus distance from the Cu electrode. Color coding is Red (O), grey (C), yellow (Cu), green (Cl), purple (K), blue (N) and white (H). ΔU, potential difference between two electrodes.

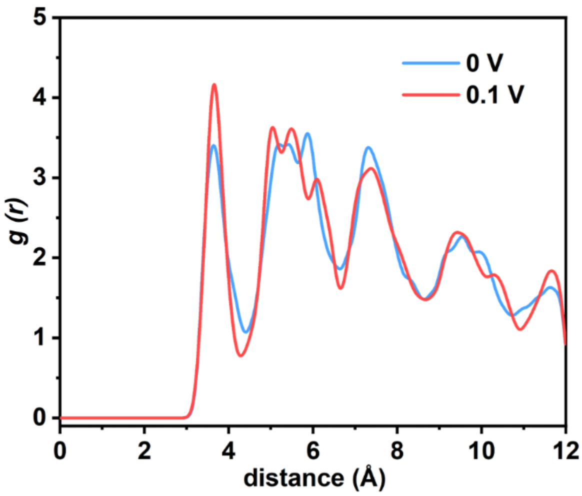

Fig. S15 Radial Distribution Functions (RDF) between Cl and Cu at different electrode potential.

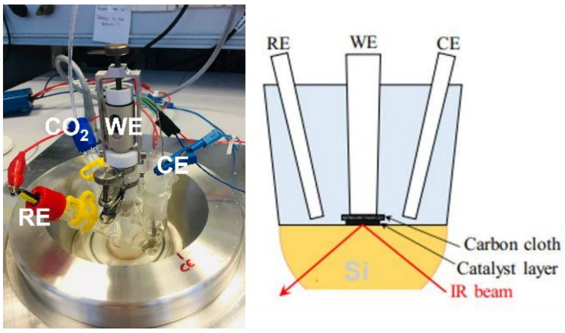

Fig. S16 Schematic illustration of the custom-made in situ electrochemical ATR setup. The cell consists of a spectroscopic bottom section with the ATR prism secured by a prism holder and a connecting plate, which links to the electrochemical glass cell. The glass cell features a gas inlet, a working electrode with an adjustment screw, a reference electrode, and a counter electrode.

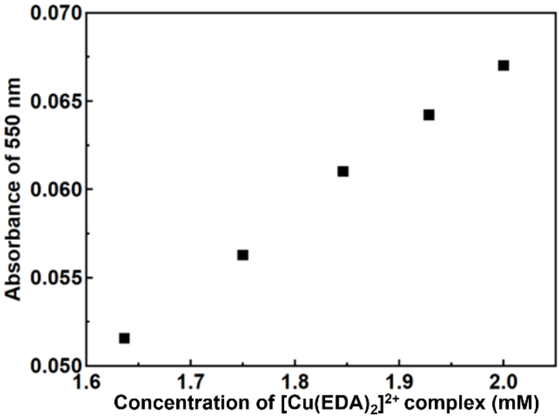

Fig. S17 Calibration curve for UV-Vis spectrophotometric determination of Cu-EDA complex in 0.1 M EDA-KCl aqueous solution (λ max = 550 nm).

Supplementary Table 1 Comparison of onset potentials versus Ag/AgCl and OCP in 0.1 M K*A* (*A* = Cl^-, NO_3^-, and ClO_4^-) supporting electrolytes.

Electrolyte	Onset potential	
	$V_{Ag/AgCl}$	V_{OCP}
KCl	-0.24	0.01
KNO_3	-0.12	0.06
$KClO_4$	-0.10	0.09

Supplementary Note 1: Electronic setting and molecular position calculation of simulation system

The SPC/E water model was employed to model the aqueous environment, with the SHAKE algorithm used to constrain the stretching mode of water molecules.[1] Periodic boundary conditions (PBC) were applied in the x and y directions, while the z-direction was

fixed. In the z-direction, the liquid phase was confined by two Cu (111) surface slabs, each with dimensions of 62.6 Å × 41.0 Å, placed on opposite sides of the simulation box. The atomic positions of the Cu (111) electrodes were frozen throughout the simulation to maintain a rigid electrode surface. A time step of 1 fs was used in the simulations. Bulk simulations were performed for all investigated systems to determine their density. Periodic boundary conditions were applied in all directions. During the initial equilibration phase, no external potential was applied, and the atomic charge of all Cu atoms was set to zero. The charge settings of anions (Cl^-, NO_3^-, ClO_4^-), cations (K^+) and CO_2 are obtained according to the reference files of force field parameters in the calculation method. The charge of CO_2-EDA was determined using the QEq charge model via the AuToFF method, with a charge scaling coefficient set to 0.01.[2] To prevent the formation of energy hotspots at the start of the simulation, an energy minimization step was performed before each simulation. This was followed by a 2 ns preliminary equilibration in the NVE ensemble, ensuring the system reached a thermodynamically stable interfacial structure, which was required for the subsequent production simulations.

The density distribution of the particles in the z direction is analyzed by the chunk/atom parameter setting. The method involves using the LAMMPS command compute chunk/atom to calculate the spatial distribution of a specific group of atoms (e.g., CO_2 molecules) along the z-axis.

Supplementary Note 2: Evaluation of excess energy consumed to overcome overpotential barriers

To assess the excess energy consumed to overcome overpotential barriers, we integrated the overpotential relative to open circuit potentials from DEMS with the FE during CO_2 release, we determined that initiating CO_2 release in the K*A* + EDA-CO_2 system required an excess energy consumption is expressed in terms of energy per mole of CO_2 released (in $kJ_e/_{molCO2}$) as follows:

$$W_{excess} = [F(V_{\eta}\text{-}V_{ocp})]/\eta_e$$

where F is the Faraday constant (96485 C/mol), V_{η} is the applied overpotential (in $V_{Ag/AgCl}$), V_{ocp} is the open circuit potential (in $V_{Ag/AgCl}$) and η_e is the electron utilization efficiency. The electron utilization quantifies the number of CO_2 molecules desorbed for every electron spent on CO_2 release.

The energy savings are calculated as the difference between the W_{excess} in the K*A* + EDA-CO_2 system and that in the KCl + EDA-CO_2 system.

References

[1] Martínez, L., Andrade, R., Birgin, E.G. & Martínez, J.M. PACKMOL: A Package for Building Initial Configurations for Molecular Dynamics Simulations. Journal of Computational Chemistry 30, 2157-2164 (2009)
[2] Wang, C., Liao, K., Wang, Z., Wang, Y. & Gong, K. AuToFF Program. (Version, 2023).
